# Flow Cytometry for Diagnosis of Primary Immune Deficiencies—A Tertiary Center Experience From North India

**DOI:** 10.3389/fimmu.2019.02111

**Published:** 2019-09-11

**Authors:** Amit Rawat, Kanika Arora, Jitendra Shandilya, Pandiarajan Vignesh, Deepti Suri, Gurjit Kaur, Rashmi Rikhi, Vibhu Joshi, Jhumki Das, Babu Mathew, Surjit Singh

**Affiliations:** Allergy Immunology Unit, Department of Pediatrics, Advanced Pediatrics Center, Post Graduate Institute of Medical Education and Research, Chandigarh, India

**Keywords:** flow cytometry, recent advances, clinical applications, immune dysregulation, immune deficiencies, primary immunodefciencies

## Abstract

Flow cytometry has emerged as a useful technology that has facilitated our understanding of the human immune system. Primary immune deficiency disorders (PIDDs) are a heterogeneous group of inherited disorders affecting the immune system. More than 350 genes causing various PIDDs have been identified. While the initial suspicion and recognition of PIDDs is clinical, laboratory tools such as flow cytometry and genetic sequencing are essential for confirmation and categorization. Genetic sequencing, however, are prohibitively expensive and not readily available in resource constrained settings. Flow cytometry remains a simple, yet powerful, tool for multi-parametric analysis of cells. While it is confirmatory of diagnosis in certain conditions, in others it helps in narrowing the list of putative genes to be analyzed. The utility of flow cytometry in diagnosis of PIDDs can be divided into four major categories: (a) Enumeration of lymphocyte subsets in peripheral blood. (b) Detection of intracellular signaling molecules, transcription factors, and cytokines. (c) Functional assessment of adaptive and innate immune cells (e.g., T cell function in severe combined immune deficiency and natural killer cell function in familial hemophagocytic lymphohistiocytosis). (d) Evaluation of normal biological processes (e.g., class switching in B cells by B cell immunophenotyping). This review focuses on use of flow cytometry in disease-specific diagnosis of PIDDs in the context of a developing country.

The term “flow cytometry” refers to evaluation of multiple cell characteristics in a flow system that delivers a single cell suspension at a defined point of measurement (1). Flow cytometry can be used for analysis of intracellular and extracellular proteins, cell sorting, apoptosis, cell proliferation, and quantification of DNA. Utility of flow cytometry in clinical studies was first described by Dr. Louis Kamentsky in the year 1965 (2). Since its first description, there have been major technological advances in the field of flow cytometry, and this technology has revolutionized the field of cell biology.

Flow cytometry is a key investigation for analysis of leucocyte subsets and function and is an essential diagnostic tool in clinical immunology. Primary immune deficiency disorders (PIDDs) are a group of inherited disorders affecting single or multiple components of the immune system, resulting in increased predisposition to infections and immune-dysregulation (3). Flow cytometry is often one of the first investigations to delineate the type of PIDD (4). In our previous review, we highlighted the progress of PIDD research in India and the spectrum of cases with PIDDs at our institute (5). In this review, we discuss the utility of flow cytometry in diagnosis and management of patients with PIDDs in context of a developing country.

## Setting Up of Flow Cytometry Facility at Our Center

**Figure 1:**
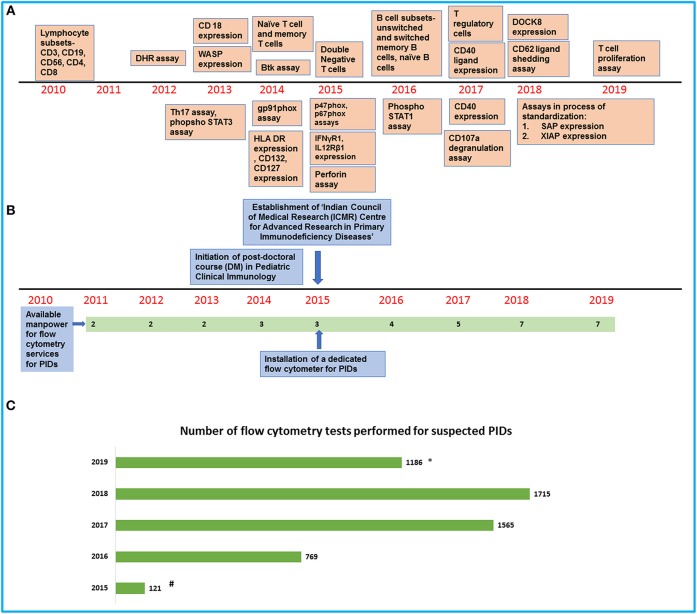
An overview of development of flow cytometry services for PIDs at our center. **(A)** Timeline showing the establishment of flow cytometry tests in the laboratory. **(B)** Timeline showing the increase in manpower and support for the laboratory services. **(C)** Bar graph showing the number of flow cytometry tests for PIDs performed in our laboratory in the last 5 years (#Data available from August 2015 to December 2015/*Data available from January 2019 to July 2019).

- We have been performing flow cytometry for clinical and research work in PIDDs on a dedicated Beckman Coulter, two-laser, six-color platform (*Navios*) (Figure 1). This is a robust and user-friendly instrument but is limited by the number of parameters that can be studied in a single tube.
- Majority of samples that we process are obtained from patients who visit our institute and these samples are processed the same day.
- Our laboratory is situated in close proximity to the clinical service areas. This facilitates processing of samples and coordination between clinical and laboratory personnel.
- However, as we are a designated Center for Advance Research in PIDDs under the Indian Council of Medical Research, we also receive samples from other parts of India (Figure 1).
- These samples are dispatched by commercial courier and usually reach our laboratory within 48–72 h. All such couriered samples are accompanied by a transport control.
- In our experience, these samples do not deteriorate if processed within 48–72 h of having been drawn but we face difficulty during summer months (May–June) when the blood often gets hemolyzed. Assays designed to estimate lymphocyte or neutrophil function (e.g., Dihydrorhodamine 123 assay) are more likely to be jeopardized due to delays in transportation than assays for estimation of cell surface molecules. We also routinely use CD45 as a gating marker and 7-aminoactinomycin D (AAD) to differentiate between live and dead cells. Abnormal results are interpreted in relation to results obtained for travel control.
- We usually follow the recommended protocols for processing samples but have made some improvisations based on our experience and keeping in mind cost constraints. We carefully titrate each fresh lot of antibodies and use the antibody concentration with the highest signal-to-noise ratio. This is often much less than the volume recommended by the manufacturer.
- All samples are processed with appropriate controls. However, this does add to laboratory costs.
- Before acquisition of samples, we run quality control (QC) beads (Flow check beads for Navios) for verification of optical alignment and fluidics stability. This can be checked by calculating coefficient of variation in different detectors. Other set of fluorospheres (Flow set) are also used on a regular basis to standardize light scatter intensity and fluorescent intensity. Stabilized leucocytes with known quantity of surface antigens (Immuno-Trol cells) are used to verify monoclonal antibody performance, sample staining (lysis), and analysis. If coefficient of variation is out of range, we clean the instrument again and rerun the quality control beads. If QC requirements are not met, we withhold processing of samples.
- For each lot of antibodies, we perform compensation in a multicolor experiment. This is achieved using cells containing mutually exclusive population of same fluorochromes. Compensation is re-established after any change in hardware, laser alignment, and change in filters or optics.

## Role of Flow Cytometry in Diagnosis of Combined Immune Deficiency (CID)

### Severe Combined Immune Deficiency (SCID)

SCID comprises a group of disorders that predominantly affect T cell development and function. In addition to T cell defects, there may be impaired B cell and/or NK cell development (6).

#### Immunophenotyping of Lymphocyte Subsets to Classify SCID

A preliminary lymphocyte subset analysis by flow cytometry that includes markers for B lymphocytes (CD19), T lymphocytes (CD3), and natural killer cells (CD56/16) is the first-line investigation for SCID, and it helps in identifying the subtypes of SCID (7–13) (Table 1, Supplementary Figure 1A). Isolated CD4 lymphopenia has been described with MHC Class II deficiency (*RFXANK, CIITA, RFXAP*) and hypomorphic *RAG* variants (14). HLA-DR expression by flow cytometry would also be decreased in patients with MHC Class II deficiency. Isolated CD8 lymphopenia with preserved CD4 counts can be seen with *TAP1, TAP2*, and *ZAP70* defects (15).

**Table 1:** Immunophenotyping in severe combined immune deficiency (SCID) with associated genetic defects.

	**Lymphocyte phenotype**	**Associated genetic defects**	**Comments**
I.	T–B–NK– SCID	*ADA, PNP*	Accumulation of toxic metabolites inhibits DNA synthesis and repair and leads to severe lymphopenia (7).
II.	T–B-NK+ SCID	*RAG1, RAG2, DCLRE1C, LIG4, NHEJ1*	Defects in somatic recombination result in decreased or absent T and B lymphocytes (8).
III.	T–B+NK– SCID	*IL2RG, JAK3*	T-B+NK- SCID results from defects in common gamma chain that is required for normal development of T and NK cells (9). Analysis of surface expression of CD132 may also help in identifying the defect (10).
IV.	T–B+NK+ SCID	*IL7 R*, CD3δ, CD3ε and CD3ζ	Reduced surface expression of CD127 on T cells can help in classifying SCID (11).
V.	Omenn syndrome	*RAG1, RAG2, DCLRE1C, ADA, LIG4, IL2RG, IL7R*, DiGeorge syndrome	Reduced naïve T cells (CD3+45RA+45RO−), elevated memory T cells (CD3+45RA-45RO+), and increased expression of HLA DR on T lymphocytes are noted in Omenn syndrome (12). T cell receptor Vβ repertoire analysis shows skewed Vβ usage indicating oligoclonality (13).

Normal numbers of CD3 counts can be seen in patients with SCID with associated Omenn syndrome (OS) or maternal T cell engraftment. Estimation of naïve T cells (CD45RA+ CD45RO–CD62L+) and memory T cells (CD45RO+CD45RA–CD62L–) in CD4+ and CD8+T lymphocyte populations is helpful in these situations as naïve T cell population is grossly decreased (16) (Supplementary Figures 1B,C).

Surface expression of common γ chain (CD132) and interleukin receptor 7α chain (CD127) on monocytes and T lymphocytes, respectively, can also be utilized to characterize X-linked SCID and IL7R deficiency, respectively (17) (Supplementary Figures 1D,E).

#### Functional Assays

Phosphorylation of downstream signal transducer and activator of transcription (STAT) is impaired in cases of defects with *IL2RG* and *JAK3*. Expression of phosphorylated STAT3 or STAT5 in the lymphocytes after stimulation with IL-2 and IL-21 is low in these patients as compared to normal controls (4).

#### Estimation of Lymphocyte Proliferation

Flow cytometry is also used for assessment of T cell proliferation in response to antigens or mitogens using carboxy fluorescein diacetate succinimidyl ester (CFSE) or cell trace dyes that integrate with intracellular proteins of the cell. When T lymphocytes are stimulated with mitogens [e.g., phytohemagglutinin (PHA)] or anti CD3 with CD28, the cells divide and the dye gets apportioned equally between two daughter cells. Patients with SCID show impaired T cell proliferation and decreased response to the dye after stimulation with PHA. Other dyes like Cell Trace Violet and CellTrace Far Red have also been used for lymphocyte proliferation studies (18).

#### Assessment of Radiosensitive Forms of SCID

Radiosensitivity flow assay is one of the newer techniques that help in functional assessment of patients with radiosensitive forms of SCID (e.g., *DCLRE1C, PRKDC, LIG4, NHEJ1*, and *NBS1*) (19). We have not standardized this in our laboratory yet.

#### Laboratory Work Flow for SCID at Our Center

In a suspected case of SCID or other CID, we first perform lymphocyte subset analysis using a limited panel of four antibodies, i.e., CD45, CD3, CD19, and CD56. Lymphocytes are gated using SSc vs. CD45 and different subsets are estimated. In patients with SCID with completely absent T cells, we do not assay naïve or memory T cell population. When T cells are present in reduced or normal numbers in a suspected case of SCID or CID, we perform T cell subset analysis using CD3, CD4, CD8, CD45RA, and CD45RO antibodies to estimate the naïve and memory T cells. As we have been getting reasonable results with this antibody cocktail, we have not considered addition of CD62L for processing these samples (20).

HLA-DR estimation on CD3+ T lymphocytes is performed along with naïve and memory T cell estimation in patients with suspected OS. We are not in a position to perform Vβ repertoire analysis in these patients as it is prohibitively expensive.

T cell function is analyzed using CFSE staining. Depending on the immune phenotype and family history we perform expression of IL-2RG on monocytes if the phenotype is T**–**B+NK**–** or if there is strong suspicion of X-linked recessive inheritance. Similarly, in patients with T**–**B+NK+ SCID, we perform IL-7R (CD127) staining. We have erroneously diagnosed IL7R deficiency when CD127 expression was checked on gated lymphocytes and found to be low. This is due to internalization of IL7R during episodes of severe lymphopenia and also due to the fact that if T cells are decreased, gating lymphocytes for IL7R expression is not an appropriate strategy. We have now developed protocols for CD127 expression on CD19+ B lymphocytes.

#### Clinical Correlate

Flow cytometry also helps in monitoring of engraftment of T cells post-hematopoietic stem cell transplantation in patients with SCID (Supplementary Figure 2). We have used flow cytometry for antenatal screening of SCID by measurement of lymphocyte subsets in cord blood samples, especially when amniocentesis or chorionic villous sampling is not possible, and genetic basis of SCID is not available.

### DOCK8 Deficiency

*DOCK8* defect is an autosomal recessive form of CID characterized clinically by severe cutaneous viral infections such as warts or molluscum contagiosum. Laboratory investigations may reveal eosinophilia and increased serum levels of IgE. Immunological features include low T and B cell numbers, decreased levels of serum IgM, and impaired functional antibody response (21).

DOCK8 is an intracellular protein expressed in myeloid and lymphoid lineages (22). Intracellular staining of DOCK8 in lymphocytes by flow cytometry can be used to recognize patients with *DOCK8* defect and also monitor the expression of DOCK8 in various cell lineages following HSCT in patients with this defect (23).

#### Laboratory Work Flow for DOCK8 at Our Center

In a suspected case of DOCK8 deficiency, we perform this assay on lymphocytes and neutrophils. As fluorochrome-labeled anti-DOCK8 is presently not available in India, we use a custom-designed antibody labeled with a chosen fluorochrome. This is technically more difficult to standardize.

### Hyper-IgM Syndrome

Hyper-IgM syndromes are inherited disorders that mainly affect somatic hypermutation and B cell class switch recombination (24). Serum IgM levels of affected patients may be normal or elevated but IgG and IgA levels are usually decreased.

X-linked Hyper-IgM syndrome occurs due to defect in *CD40L* that encodes for CD40 (CD154) present on activated T cells. The assay is usually performed along with CD69 or CD25 staining of lymphocytes to confirm lymphocyte activation status. Increased expression of CD69 or CD25 along with decreased or absent expression of CD154 on activated lymphocytes is suggestive of CD40L defect (25) (Supplementary Figure 3). In our experience, flow cytometry may not give a clue in all patients with CD40L defect. As expression of CD40L can be normal in 5–10% cases, staining with CD40-muIg can be used in these situations. Autosomal recessive hyper-IgM syndrome due to CD40 defect can also be identified by flow cytometry by analyzing expression of CD40 in B cells.

#### Laboratory Work Flow for Hyper IgM at Our Center

In our center, we study CD40L (CD154) expression by flow cytometry on activated CD4+/CD69+ helper T cells after stimulation with phorbol myristate acetate (PMA) and ionomycin. Percentage expression and median fluorescence intensity of CD40L on activated dual positive CD4+/CD69+ helper T cells is compared with age- and sex-matched healthy controls.

### Wiskott-Aldrich Syndrome (WAS)

WAS is an X-linked recessive condition characterized by eczema, thrombocytopenia (with platelets that are characteristically small in size), and CID (26). The *WAS* gene encodes for a 502-amino acid protein (WASp) that contributes to cell motility, actin polymerization, and apoptosis (27). The WASp antibody is directed against WAS protein that is evaluated both on lymphocytes and monocytes.

Flow cytometry plays an important role in detection of WASp through intracellular staining, after fixation and permeabilization of cells (28).

#### Laboratory Work Flow for WAS at Our Center

In patients with WAS, we perform intracellular staining assay of WAS. We use CD45 and fluorochrome-labeled WAS antibody for this assay. Lymphocytes, monocytes, and neutrophils are gated on CD45 vs. SSc, and expression of WAS protein is checked in each of these leucocyte subsets. The presence or absence of protein is determined by calculating Stain Index (SI) ($\text{SI}\;=\;\frac{Median\;fluorescence\;intensity\;(MFI)\;of\;Stained\;cells}{Median\;Fluorescence\;Intensity\;of\;unstained\;cells}$) of controls as well as patient samples and then SI ratio (**SI ratio** **=** $\frac{SI\;of\;patient}{SI\;of\;control}$) is calculated for each patient. We consider an SI ratio <0.65 on gated lymphocytes to be suggestive of WAS. The SI ratio of patient vs. control remains uniform irrespective of change in MFI, which occurs at each instance when the flow cytometer is recalibrated. We have performed 127 WASp assays from January 2016 to May 2019. In 76 suspected cases, the SI of the test was compared with that of single control/case. Similarly, for 10 cases, the SI of test was compared to the average SI of two controls for each case. Lately, in 41 suspected cases, we have compared the SI of test with an average SI of 3 controls/case. A ratio of 0.65 was determined as a cutoff from these comparisons.

#### Clinical Correlate

Flow cytometry often provides the first clue in identification of WAS in males being worked up for persistent thrombocytopenia. Normal WASp expression, however, does not rule out WAS as the protein may be non-functional. In such cases, genetic workup is warranted. Flow cytometry can also be used to monitor patients with WAS who have undergone HSCT and also to check for carrier status of females who would typically show a bimodal expression of WASp due to lyonization (Supplementary Figure 4).

## Role of Flow Cytometry in Humoral Immune Deficiencies

### X-Linked Agammaglobulinemia (XLA)

XLA is an X-linked recessive antibody deficiency due to mutations in the *BTK* gene. Investigations reveal profound hypogammaglobulinemia with decreased or absent peripheral B cells and reduced BTK expression in monocytes on flow cytometry (29). Female carriers of XLA show a bimodal expression of BTK protein (30). In some patients, it becomes necessary to perform genetic analysis and correlate with the BTK flow analysis, as some missense mutations may show near-normal levels of BTK protein expression (31) (Supplementary Figure 5).

#### Laboratory Work Flow for XLA at Our Center

For patients with suspected XLA, we perform CD3/CD19/CD56 lymphocyte subset assay by gating lymphocytes on CD45 vs. SSc. Btk protein expression analysis is carried out on monocytes in patients with low B cell count (<2%). CD14 antibody is used for labeling monocytes for Btk expression. A control sample is always run for comparison. Both percentage and median fluorescence intensity (ΔMFI) are measured in patients and control.

### Common Variable Immune Deficiency (CVID)

The term common variable immune deficiency (CVID) refers to a heterogeneous group of diseases defined by low IgG and IgA or IgM deficiency with a decreased antibody response to vaccination (32). Unlike XLA, patients with CVID usually do not have decreased number of B cells. However, most patients with CVID have an abnormal B cell differentiation and reduced memory B cells (33). Memory B cell subsets that are commonly assayed in CVID include class-switched memory B cells (CD19+CD27+IgM–IgD–), marginal zone-like B cells (CD19+CD27+IgM+IgD+), plasmablasts (CD19+CD20−IgM-CD38^hi^CD27+), transitional B cells (CD19+CD27−CD24^hi^CD38^hi^IgM^hi^CD10+), and CD21– and CD21+ B cells (34). These can be evaluated by flow cytometry (Supplementary Figure 6).

A large number of monogenic defects have been described in patients with CVID. These include defects in *CD19, CD81, CR2, MS4A1, ICOS, CTLA4, LRBA, NFKB1, NFKB2, TNFRSF13C, PIK3CD*, and *PIK3R1* (35). However, more than 80% of patients with CVID do not appear to have any monogenic basis for their disease (36). Expression of CD19, CD81, CD21, CD20, ICOS, and BAFF-R can be estimated by flow cytometry. ICOS is detected on activated T cells and BAFF-R is detected on B cells. Cytotoxic T lymphocyte-associated protein 4 (CTLA-4) is a costimulatory molecule. It is expressed on activated T cells and binds to CD28 on B cells. CTLA-4 is essential for functioning of regulatory T cells (37). CTLA-4 expression on flow cytometry is studied on mitogen-stimulated CD4+ T lymphocytes (38). Intracellular expression of Lipopolysaccharide Responsive Beige-like Anchor Protein (LRBA) can be assessed by flow cytometry in monocytes, T cells, and NK cells.

#### Laboratory Work Flow for CVID at Our Center

We perform B cell immunophenotyping for naïve, switched, and unswitched B lymphocytes in patients with suspected CVID using CD19, CD27, sIgD, and sIgM antibodies. We do not routinely estimate transitional B cells or CD21 low B cells, although this would be useful in defining distinct subsets of CVID patients.

#### Clinical Correlate

Secondary hypogammaglobulinemia can occur due to drugs or loss of immunoglobulin in conditions such as nephrotic syndrome and protein losing enteropathy. Clinicians often face a dilemma when patients with autoimmune disorders who are receiving steroids turn out to have a low IgG level. This can be a manifestation of CVID or may represent secondary hypogammaglobulinemia due to glucocorticoids. Identification of reduced switch memory B cell patients with low IgA concentration has been reported to be useful in differentiating CVID from secondary hypogammaglobulinemia due to glucocorticoids (39).

## Role of Flow Cytometry in Diseases With Immune Dysregulation

### Hemophagocytic Lymphohistiocytosis (HLH)

HLH is a life-threatening disorder due to excessive activation of macrophages and cytotoxic lymphocytes resulting in a cytokine storm. HLH is composed of two major subtypes: primary HLH due to genetic defects and secondary HLH that is associated with an underlying predisposing condition such as an infection, malignancy, or rheumatologic disease (40). Primary HLH results from genetic defects affecting function of NK and cytotoxic CD8+T cells. The most common genetic defect identified is *PRF1* that encodes for perforin, a cytolytic enzyme released by NK cells or CD8+ T cells for killing of intracellular pathogens. Defects in *UNC13D, STXBP2*, and *STX11* (41–43) that are involved in cytotoxic lymphocyte degranulation also lead to primary HLH. Flow cytometry helps in measurement of perforin expression and evaluation of CD107a degranulation in NK and CD8+ T cells (44, 45) (Supplementary Figure 7). Other defects like Chédiak–Higashi syndrome, Griscelli syndrome Type 2, and Hermansky-Pudlak syndrome Type 2 also have defective degranulation. X-linked lymphoproliferative (XLP) syndrome is a rare disorder characterized by profound hypogammaglobulinemia, Epstein–Barr virus (EBV)-induced lymphoproliferation, and fatal hemophagocytosis. Patients with XLP1 have a defect in the SH2 domain of a SLAM-associated protein (SAP). XLP2 is due to a defect in *XIAP*, which is an X- linked inhibitor of apoptosis gene. Expression of both XIAP and SAP can be detected by intracellular staining on flow cytometry (46) (Supplementary Table 1).

#### Laboratory Work Flow for HLH at Our Center

In patients with suspected HLH, we carry out perforin expression by flow cytometry on CD56+ NK cells after intracellular staining. When NK cells are markedly reduced, we estimate the perforin expression on cytotoxic T cells (CD3+CD8+). If perforin expression is found to be normal, granule release assay (CD107a) is performed on PBMCs by stimulating the cells with PMA and ionomycin and Golgi stop (monensin). We also perform CD107a assay in patients with Chédiak–Higashi syndrome and Griscelli Type 2 syndrome.

### Autoimmune Lymphoproliferative Syndrome (ALPS)

ALPS is characterized by lymphoproliferation, multilineage cytopenias, and an increased risk of B cell lymphoma (47). Patients with ALPS usually have defects in FAS and FASL that mediate apoptosis of lymphocytes. Defective apoptosis of developing lymphocytes in thymus results in an increase of double-negative T cells (CD3+TCRαβ+CD4–CD8–) (48) (Supplementary Figure 8). Annexin-V^+^/propidium iodide (PI2) staining helps in detecting reduced apoptosis in this disorder (49).

#### Laboratory Work Flow for ALPS at Our Center

We estimate double-negative T (DNT) cells in patients with suspected ALPS using CD3, TCRαβ, CD4, and CD8 antibodies. CD4 and CD8 antibodies are tagged with the same fluorochrome. Percentage of DNTs >1.5% of all lymphocytes or 2.5% of CD3+ T lymphocytes is considered abnormal.

### Immune Dysregulation, Polyendocrinopathy, Enteropathy X-Linked (IPEX) Syndrome

IPEX syndrome is a rare x-linked recessive monogenic autoimmune disorder resulting from a mutation in the gene *FOXP3*. Patients present with eczematous dermatitis, enteropathy, and an endocrinopathy (usually diabetes mellitus or hypothyroidism) (50). Most patients with this disorder have reduced CD4+CD25+CD127^low^ Treg or CD4+CD25+FOXP3+ cells. Normal numbers of CD4+CD25+CD127^low^ cells are found in case of hypomorphic *FOXP3* mutation (51). CD25 deficiency is an autosomal recessive defect that has similar clinical manifestations to that of IPEX syndrome. CD25 deficiency is caused by pathogenic variants in the *IL2RA* gene that codes for the α subunit (CD25) of IL2 receptor complex. IL2 α chain with β (CD122) and γ (CD132) subunits forms the high-affinity IL2 receptor. CD25 is present on the surface of T regulatory cells that help maintaining immune homeostasis (52).

#### Laboratory Work Flow for IPEX Syndrome at Our Center

We analyze T regulatory cells by surface staining with CD4+CD25+CD127+ or by analysis of intracellular FOXP3 expression in suspected cases of IPEX syndrome.

## Role of Flow Cytometry in Phagocytic Defects

### Chronic Granulomatous Disease (CGD)

CGD results from a dysfunctional NADPH oxidase activity leading to defective oxidative burst in neutrophils (53). The NADPH oxidase complex has six subunits—two cell-membrane-bound proteins (gp91phox and p22phox, encoded by *CYBB* and *CYBA* genes) and four cytosolic components (p47phox, p67phox, p40phox, and cybc1 encoded by *NCF1, NCF2, NCF4*, and *CYBC1* genes) (54, 55). Patients with CGD have reduced or absent NADPH oxidase activity leading to reduced or no conversion of DHR into fluorescent rhodamine (56) (Supplementary Figure 9).

The ΔMFI is markedly decreased in patients with CGD. Further, flow cytometry analysis for NADPH oxidase components may be performed by surface staining of gp91phox for X-linked CGD and intracellular staining for cytoplasmic component p47phox and p67phox in patients with autosomal forms of CGD. In x-linked carriers of CGD, the DHR histogram depicts a pathognomonic double peak pattern due to lyonization (57) (Supplementary Figure 10). The DHR assay can also be performed on cord blood for screening of antenatal cases. The laboratory workflow for CGD at our center is summarized in Supplementary Figure 11.

### Leukocyte Adhesion Defect

Leukocyte adhesion defect is characterized clinically by recurrent bacterial infections with little or no pus formation. Children usually present with omphalitis and delayed separation of umbilical cord. There are three types of LAD:

**LAD I**: It is an autosomal recessive disorder caused by reduced functioning or expression of CD18, the β subunit of leukocyte β2 integrins. It is caused by mutation in the *ITGB2* (integrin b2, CD18) that encodes for β2 integrin (58). Reduced or absent expression of CD18 or CD11 in neutrophils in LAD1 can be detected by flow cytometry (Supplementary Figure 12).
**LAD II**: Patients with LAD II have abnormalities in fucosylation of cell-surface glycoprotein. Reduced or absent expression of CD15 on neutrophils is characteristic of LADII.
**LAD III**: Patients with LAD III present with recurrent infections, increased bleeding tendency, and leucocytosis. LAD III deficiency can be detected by *FERMT3* gene sequencing. Neutrophil adherence and chemotaxis are significantly decreased in these patients (59). We currently do not perform neutrophil chemotaxis assay at our laboratory.

#### Laboratory Work Up of LAD at Our Center

In suspected cases of LAD deficiency, we assess CD18, CD11a, CD11b, and CD11c expression on gated neutrophils.

## Role of Flow Cytometry in Diagnosis of Other Immune Deficiency Syndromes

### STAT3 Loss of Function

Autosomal dominant hyper IgE syndrome (HIES) results from a loss of function mutation in STAT3. STAT3 defect impairs the downstream Th17 pathway and this explains the increased frequency of infections with extracellular organisms such as *Staphylococcus aureus* and *Candida albicans* (60). Th17 lymphocyte number and pSTAT3 expression (upon IL-6 stimulation) is reduced in patients with *STAT3* defect.

#### Clinical Correlate

Clinical presentation of patients with infantile eczema can, at times, be similar to that of HIES. In such situations, the National Institutes of Health (NIH) score can be useful in differentiating the two conditions (61). Flow-cytometry-based assessment of Th17 and pSTAT3 also helps in resolving the clinical dilemma. Patients with STAT3 defect usually have a decreased Th17 number and reduced pSTAT3 expression.

#### Laboratory Work Flow of HIES at Our Center

In our center, we perform Th17 cell analysis and also study the pSTAT3 expression on peripheral blood mononuclear cells (PBMCs) after stimulation with PMA and ionomycin or IL-6.

## Role of Flow Cytometry in Diagnosis of Innate Immunity

### Mendelian Susceptibility to Mycobacterial Disease (MSMD)

Patients with MSMD have a defect in the IFN-γ/IL-12 pathway and are prone to infections with *Mycobacterium* and *Salmonella* spp. (62). Genetic defects that have been shown to result in MSMD include *IFNGR1, IFNGR2, IL-12RB1*, and *STAT1* (63). Flow cytometry helps in detection of IL-12RB1 on mitogen-stimulated T cells and both subunits of IFN-γ receptor (IFNGR1 and IFNGR2). In autosomal recessive IFN-γR1 deficiency, there is a reduced expression of IFN-γR1 (64). However, in partial autosomal dominant IFNγR1 defect, IFNGR1 is overexpressed due to impaired recycling of the receptor (65). Intracellular staining of STAT1 after stimulation with recombinant IFN-γ may help in assessment of function of IFNγR1 and IFNγR2 (66). Similarly, function of IL-12Rβ1 can be assessed by measuring phosphorylated STAT4 (pSTAT4) in lymphocytes following stimulation with IL-12 (67) (Supplementary Figure 13).

#### Laboratory Work Flow of MSMD at Our Center

In a suspected case of MSMD, we perform IFN-γR1 and IL12Rβ1 assay using flow cytometry. The IFN-γR1 assay is carried out by surface staining using anti-human CD119. Lymphocytes, monocytes, and neutrophils are gated using SSc vs. FSc, and the percentage of IFN-γR1-positive cells is estimated along with the median fluorescence intensity. In patients with complete or partial IFNγR1 deficiency, the expression is reduced. However, in patients with partial dominant IFNγR1 defect, the expression may be paradoxically increased compared to control. Percentage of CD212 is analyzed on CD3-positive cells after stimulation of PBMCs with PHA for 3 days. A significantly reduced SI is a pointer toward IL12Rβ1 defect.

## Use of Flow Cytometry in Diagnosis of IRAK4 and MyD88 Deficiency

Interleukin-1 receptor-associated kinase (IRAK4) plays an important part in Toll-like receptor and IL-1 receptor signaling. Ligand binding leads to trigger of adapter protein myeloid differentiation primary response 88 (MyD88) that augments the downstream signal induction (68). Mutation in *MYD88* or *IRAK4* impairs TLR signaling pathway. Shedding of CD62L from the surface of granulocytes occurs if the TLR signaling pathway is intact. In case of defect in MYD88 and IRAK-4, CD62L shedding is impaired and this can be assessed with the help of flow cytometry after stimulation of granulocytes with lipopolysaccharide (TLR4 ligand) (Supplementary Table 2).

### Laboratory Work Flow for MyD88/IRAK4 Deficiency at Our Center

In our laboratory, we stimulate neutrophils with PMA or LPS [such as LPS (TLR4 agonist)] and then stain these with anti-human CD62L. Patients with MyD88, IRAK4, and NEMO do not shed CD62L on stimulated neutrophils. A control sample is always necessary for this assay. We do not have facilities to see the cytokine measurements (TNF α) after stimulation with LPS.

## Flow Cytometry Assays That Are Planned to Be Developed in Our Laboratory

### Activated PI3 Kinase Delta Syndrome (APDS)

Patients with APDS syndrome may harbor heterozygous gain-of-function mutation in *PIK3CD* (APDS1) or loss-of-function mutations in *PIK3R1* (APDS2), resulting in an enhanced PI3K and downstream Akt/mTOR signaling. Premature immunosenescence and immune exhaustion involving T cells leads to an increased predisposition to infection and autoimmunity. T cell immunosenescence is associated with telomere-dependent replicative senescence of cells, which is measured by cell surface expression of CD57 (69). Senescent (CD8+CD57+) T lymphocytes accumulate in the enlarged lymph nodes (70). Flow cytometry can also be used in analysis of enhanced phosphorylation of AKT in CD3 and CD19 cells.

### Ataxia Telangiectasia

Ataxia telangiectasia (AT) is a neurodegenerative disorder characterized by conjunctival telangiectasia, radiosensitivity, cerebellar ataxia, immunological defects, and increased susceptibility to malignancy (71).

It is characterized by mutation in the *ATM* gene encoding a protein that assists in cell cycle arrest of damaged cells, recombination, apoptosis, and DNA repair. The earliest event at the double-strand break is phosphorylation of H2AX (histone) protein; measurement of γ-H2AX protein can be carried out by irradiating the cells and staining the cells with γ-H2AX antibody. Levels of γ-H2AX protein are found to be lower in patients with AT as compared to normal control, thereby making it a good diagnostic test for patients with AT (72).

## Conclusion

Flow cytometry plays a major role in diagnosis and classification of PIDDs and is often used as a first-line investigation. Expression of several surface and intracellular proteins can be reliably assessed and quantified using multicolor flow cytometry. It is also the preferred technique for studying cell signaling pathways and cell–cell interactions and for assaying lymphocyte proliferation. Recent advances in flow cytometry techniques would further facilitate the workup of patients with PIDDs.

## Author Contributions

AR, KA, JS, PV, SS, and DS drafted the manuscript. AR, KA, JS, RR, GK, VJ, JD, and BM performed the laboratory investigations. SS, AR, and PV reviewed and finalized the manuscript.

### Conflict of Interest Statement

The authors declare that the research was conducted in the absence of any commercial or financial relationships that could be construed as a potential conflict of interest.

## Supplementary Material

Supplementary Figure 1 **(A)** Lymphocyte subset analysis depicting normal lymphocyte proportions of lymphocytes in healthy control; Absent T lymphocytes and reduced NK cells in a case of T–B+NK– SCID; Reduced T lymphocytes, normal NK, and relative increase in proportion of B lymphocytes representing T–B+NK+ SCID; Absent T and B lymphocytes with increased proportion of NK cells representing T–B–NK+ SCID; Severe lymphopenia with reduced T, B, and NK cells (T–B–NK– SCID) in an infant with ADA deficiency; Lymphocytes were gated on CD45 vs. SSc and different subsets were estimated on gated lymphocytes. **(B)** Decreased total naïve T lymphocytes (CD3+CD45RA+CD45RO–), naïve helper T cells (CD3+CD4+CD45RA+CD45RO–) and naïve cytotoxic T cells (CD3+CD8+CD45RA+CD45RO–) in a case of SCID; CD3+ T cells were gated on CD3 vs. SSc and CD45RA was observed on CD3, CD4, and CD8 cells. **(C)** Increased expression of HLA DR on T lymphocytes in a patient with Omenn syndrome. **(D)** Reduced expression of CD127 on gated lymphocytes in a case T–B+NK+ SCID due to mutation in IL-7R. **(E)** Reduced expression of CD132 (common γ chain) on neutrophils, monocytes, and lymphocytes in a case of T–B+NK– SCID due to mutation in *IL2RG*.

Supplementary Figure 2 Lymphocyte subset analysis in a patient with T-B-NK+ SCID **(A)** Pre-transplant; **(B–E)** After hematopoietic stem cell transplantation. Lymphocytes were gated on CD45 vs. SSc and further subsets were analyzed on gated lymphocytes.

Supplementary Figure 3 Decreased expression of CD40L on activated T Lymphocytes (CD4+CD69+) in response to stimulation with PMA and ionomycin. PBMCs were gated on FSc vs. SSc and activated T lymphocytes were analyzed on the gated PBMCs.

Supplementary Figure 4 Expression of Wiskott–Aldrich Syndrome protein (WASp) in **(A)** a healthy control; **(B)** a patient with WAS; **(C)** after hematopoietic stem cell transplantation in patient depicted in **(B)**; Lymphocytes were gated on FSc vs. SSc.

Supplementary Figure 5 **(A)** Absent B lymphocytes and **(B)** absent Btk protein expression on gated monocytes in a patient with XLA.

Supplementary Figure 6 B cell phenotyping showing decreased class switched B lymphocytes and reduced switched memory B lymphocytes in a case of CD40 deficiency. **(A)** Control **(B)** Patient. Lymphocytes were gated on FSc. vs SSc, further B lymphocytes were gated on CD19+ to analyze the B cell subsets.

Supplementary Figure 7 Evaluation for hemophagocytic lymphohistiocytosis. **(A)** Reduced perforin expression on CD56+NK cells in a patient with HLH; lymphocytes were gated on FSc vs. SSc graph. Further, the CD56+ cells are analyzed on gated lymphocytes. **(B)** PBMCs stimulated with PMA and ionomycin and stained with CD107a show reduced expression in a patient. PBMCs were gated on FSc vs. SSc followed by CD56+ NK cells from the gated PBMCs.

Supplementary Figure 8 Increase in double-negative T cells (TCR αβ+, CD3+, CD4–, CD8–) in a patient with ALPS (FASL mutation). Lymphocytes were gated on CD45 vs. SSc graph and further DNTs were seen on CD4 CD8 vs. TCR-αβ dot plot.

Supplementary Figure 9 Dihydrorhodamine assay for chronic granulomatous disease: **(A)** Normal shift of fluorescence peak in a control. **(B)** No shift in a patient with CGD. **(C)** Bimodal expression in a female carrier of X-linked CGD. Neutrophils were gated on FSc vs. SSc plot and oxidative burst was evaluated on gated neutrophils.

Supplementary Figure 10 Flow cytometry analysis of b558 expression. **(A)** Normal expression in a control. **(B)** Patient with X-linked CGD. **(C)** Dual peaks in a carrier female. Neutrophils were gated on FSc vs. SSc plot and b558 was evaluated on gated neutrophils.

Supplementary Figure 11 Laboratory workflow for patients with chronic granulomatous disease (CGD) diagnosed at our center. ^*^DHR/gp91phox expression in the mother is performed only in cases of males with CGD.

Supplementary Figure 12 Absent expression of CD18 on gated neutrophils in a patient with LAD-1. Neutrophils were gated on FSc vs. SSc plot.

Supplementary Figure 13 **(A)** Decreased expression of IL12Rβ1 after stimulation of PBMCs with PHA; PBMCs were gated on FSc vs. SSc followed by CD3+ T cells from the gated PBMCs. **(B)** Increased expression of IFNγR1 expression on gated neutrophils in a patient with partial dominant IFNγR1 defect. Neutrophils, monocytes, and lymphocytes were gated on FSc vs. SSc plot.

Supplementary Table 1 Flow cytometry-based tests for diseases of immune dysregulation.

Supplementary Table 2 Flow cytometry-based tests for defects in intrinsic and innate immunity.
