# Supplementary figures and images for: Flow Cytometry for Diagnosis of Primary Immune Deficiencies—A Tertiary Center Experience From North India
Source: Front Immunol. 2019 Sep 11;10:2111. doi: 10.3389/fimmu.2019.02111 (PMC6749021; doi:10.3389/fimmu.2019.02111)

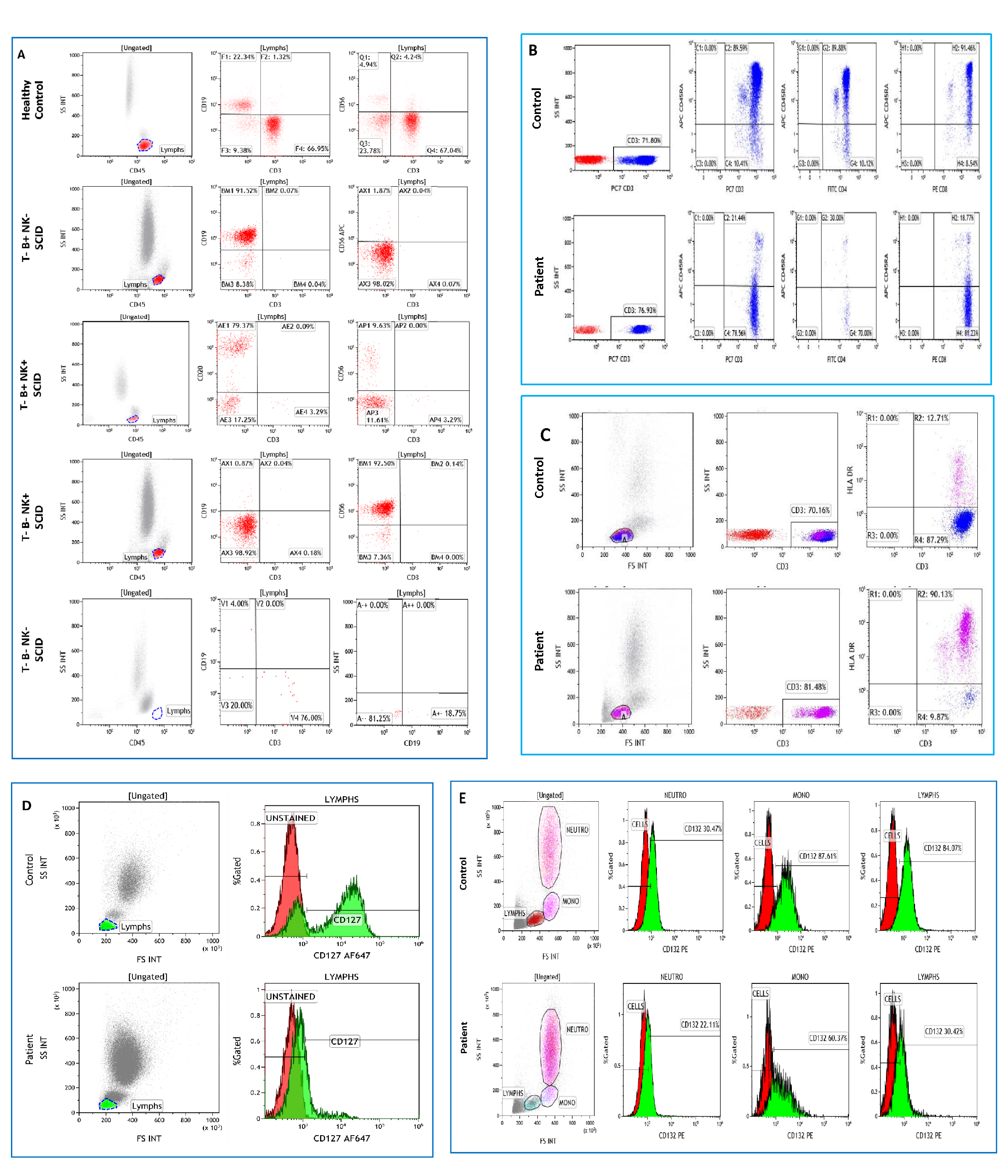

Supplement: Supplementary Figure 1 — (A) Lymphocyte subset analysis depicting normal lymphocyte proportions of lymphocytes in healthy control; Absent T lymphocytes and reduced NK cells in a case of T–B+NK– SCID; Reduced T lymphocytes, normal NK, and relative increase in proportion of B lymphocytes representing T–B+NK+ SCID; Absent T and B lymphocytes with increased proportion of NK cells representing T–B–NK+ SCID; Severe lymphopenia with reduced T, B, and NK cells (T–B–NK– SCID) in an infant with ADA deficiency; Lymphocytes were gated on CD45 vs. SSc and different subsets were estimated on gated lymphocytes. (B) Decreased total naïve T lymphocytes (CD3+CD45RA+CD45RO–), naïve helper T cells (CD3+CD4+CD45RA+CD45RO–) and naïve cytotoxic T cells (CD3+CD8+CD45RA+CD45RO–) in a case of SCID; CD3+ T cells were gated on CD3 vs. SSc and CD45RA was observed on CD3, CD4, and CD8 cells. (C) Increased expression of HLA DR on T lymphocytes in a patient with Omenn syndrome. (D) Reduced expression of CD127 on gated lymphocytes in a case T–B+NK+ SCID due to mutation in IL-7R. (E) Reduced expression of CD132 (common γ chain) on neutrophils, monocytes, and lymphocytes in a case of T–B+NK– SCID due to mutation in IL2RG. [file Image_1.tif]

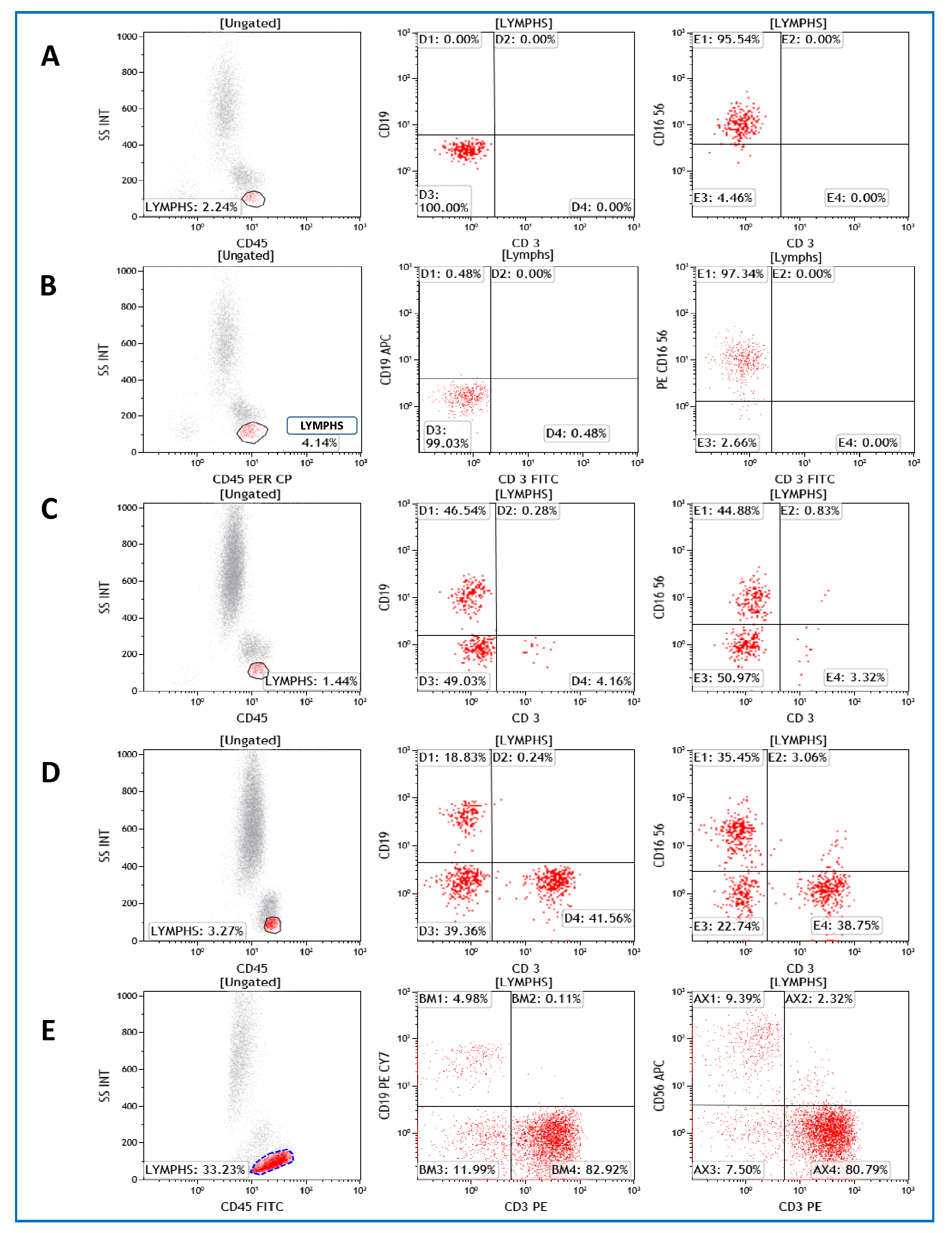

Supplement: Supplementary Figure 2 — Lymphocyte subset analysis in a patient with T-B-NK+ SCID (A) Pre-transplant; (B–E) After hematopoietic stem cell transplantation. Lymphocytes were gated on CD45 vs. SSc and further subsets were analyzed on gated lymphocytes. [file Image_2.tif]

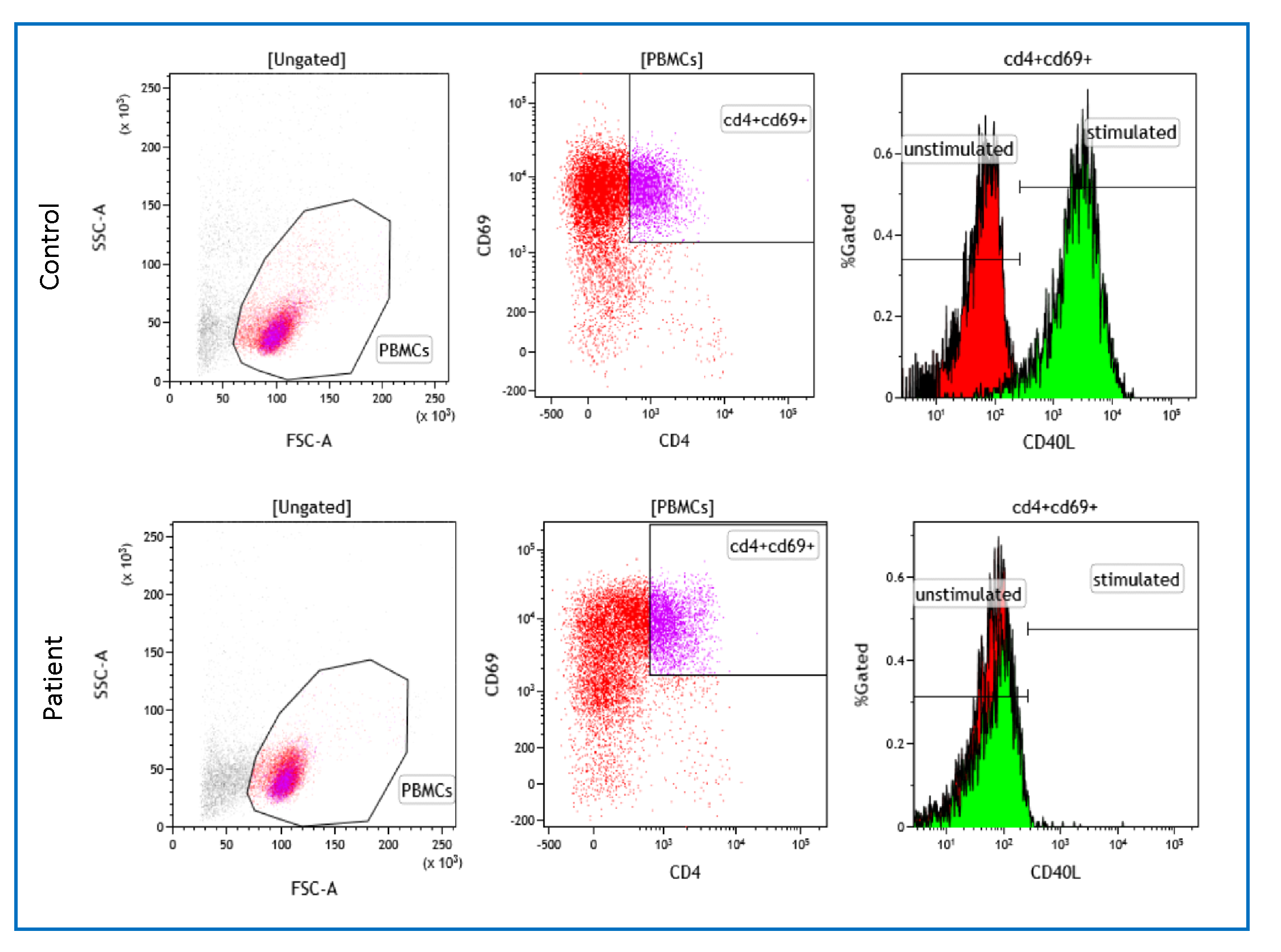

Supplement: Supplementary Figure 3 — Decreased expression of CD40L on activated T Lymphocytes (CD4+CD69+) in response to stimulation with PMA and ionomycin. PBMCs were gated on FSc vs. SSc and activated T lymphocytes were analyzed on the gated PBMCs. [file Image_3.tif]

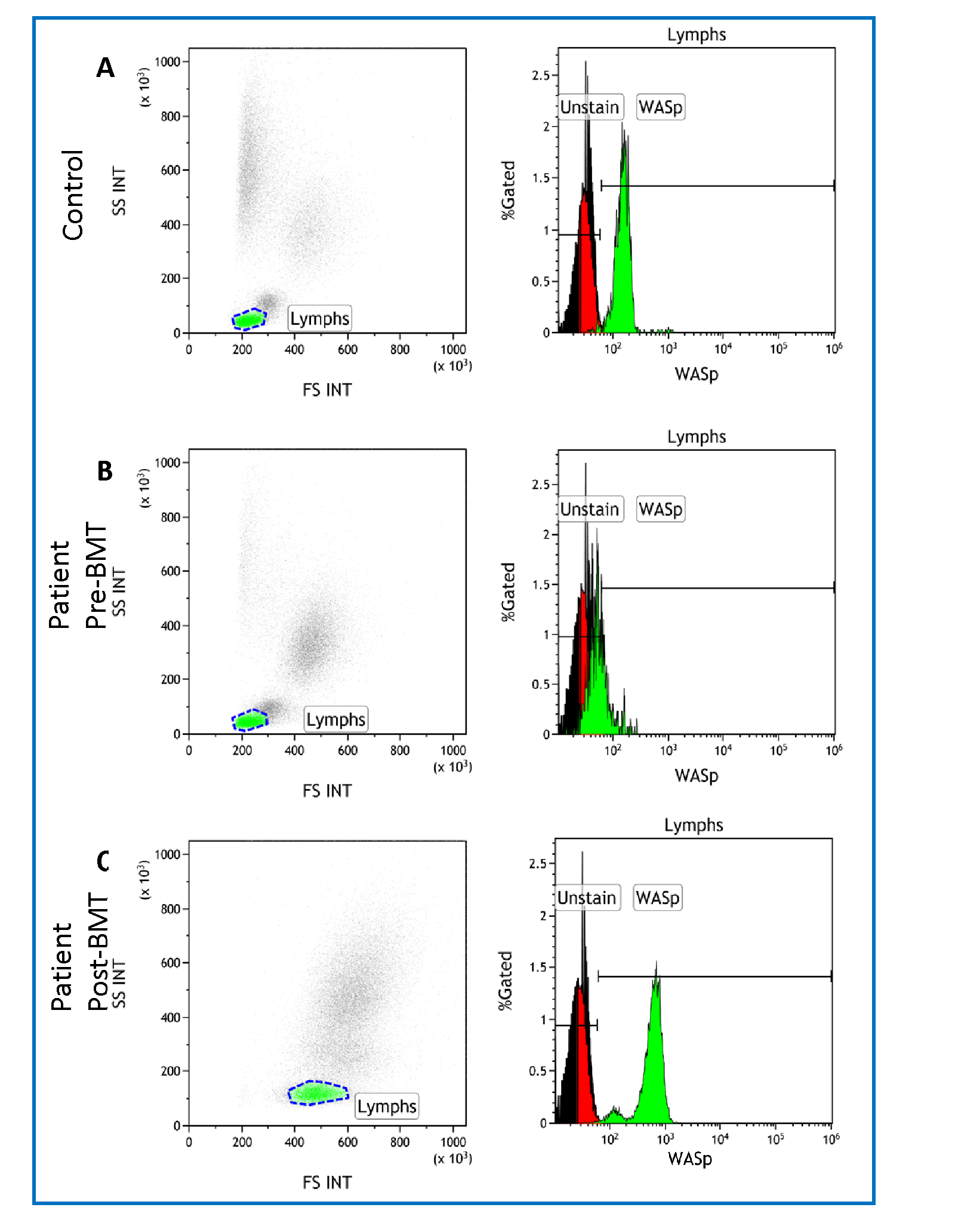

Supplement: Supplementary Figure 4 — Expression of Wiskott–Aldrich Syndrome protein (WASp) in (A) a healthy control; (B) a patient with WAS; (C) after hematopoietic stem cell transplantation in patient depicted in (B); Lymphocytes were gated on FSc vs. SSc. [file Image_4.TIF]

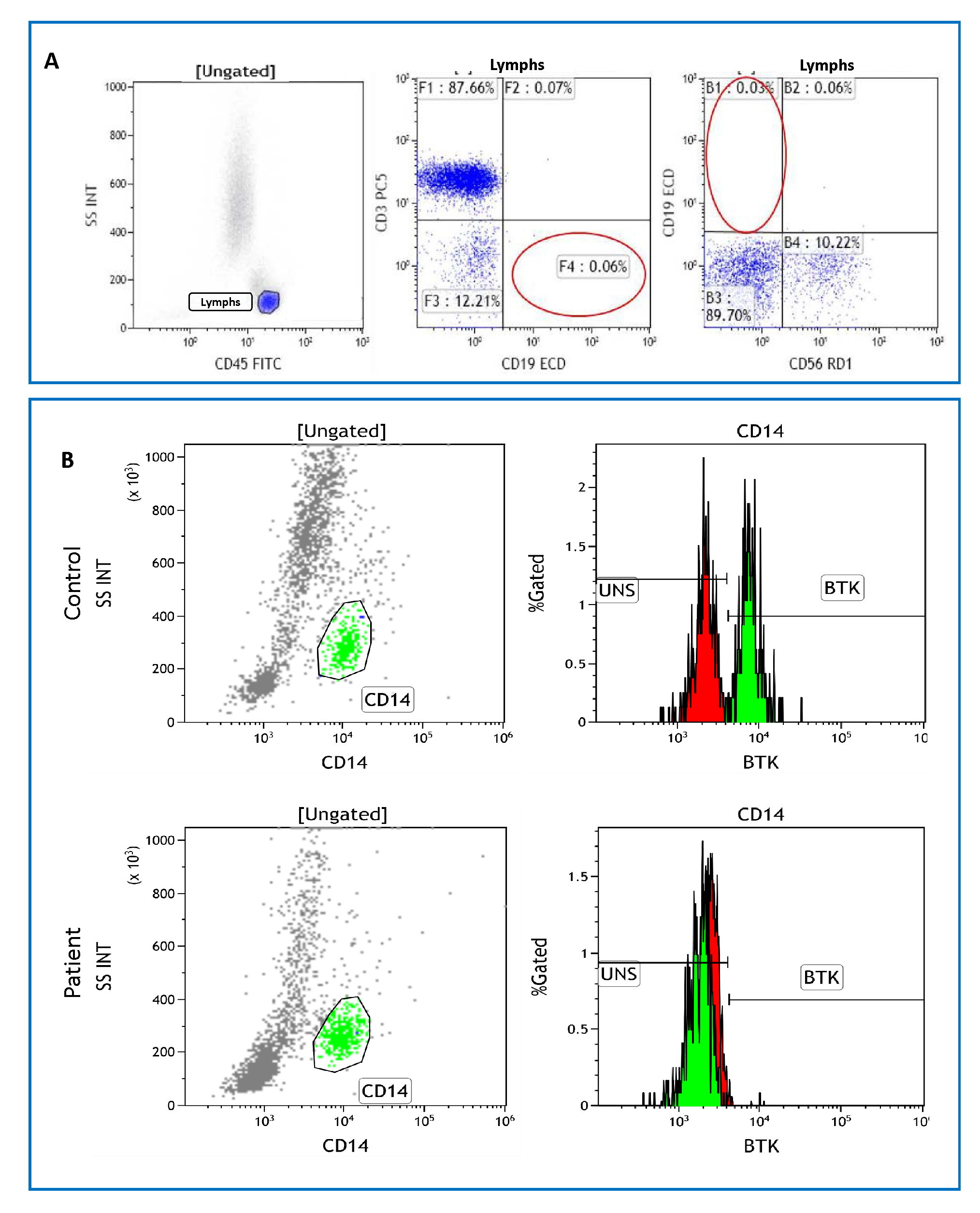

Supplement: Supplementary Figure 5 — (A) Absent B lymphocytes and (B) absent Btk protein expression on gated monocytes in a patient with XLA. [file Image_5.TIF]

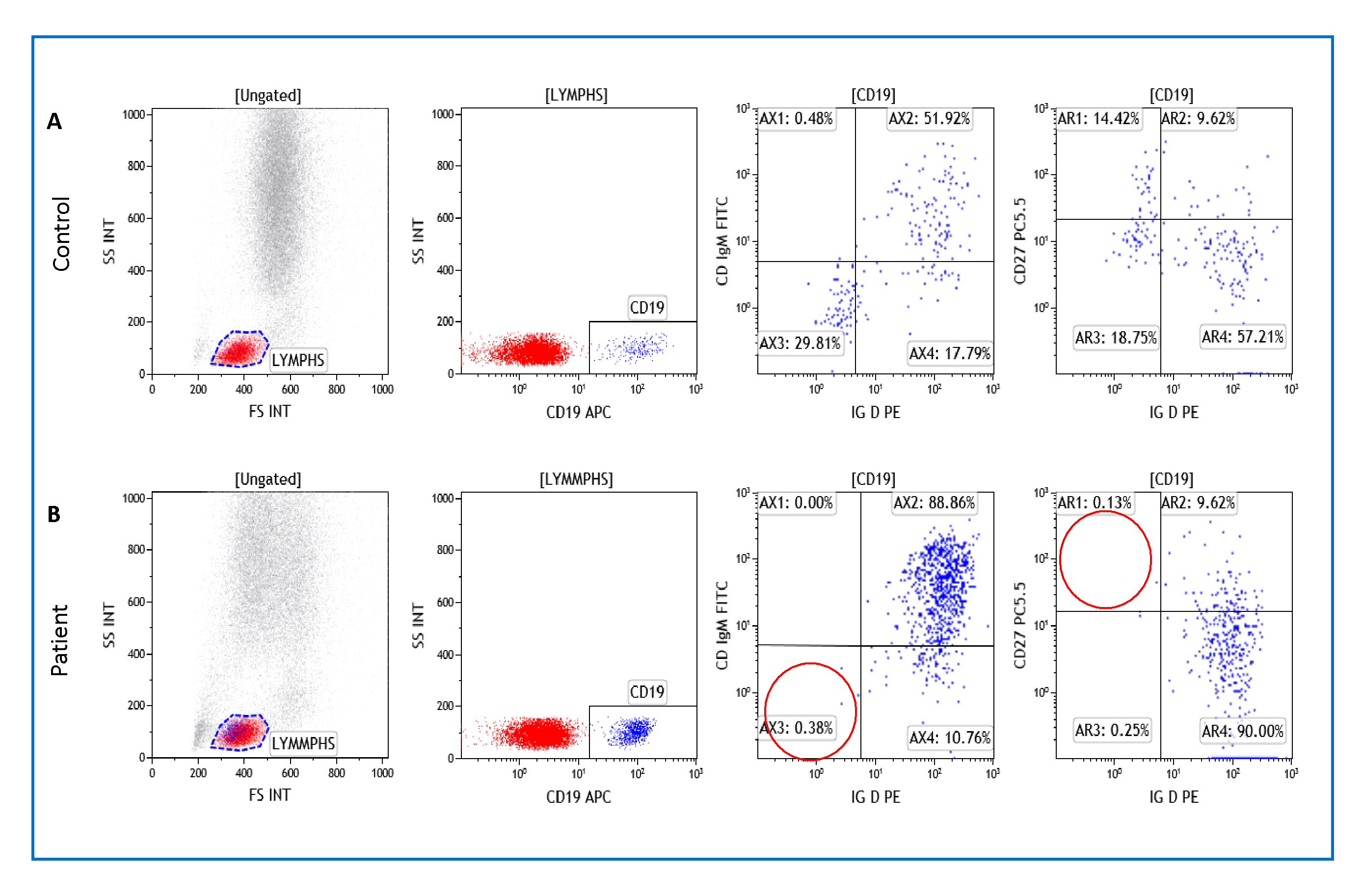

Supplement: Supplementary Figure 6 — B cell phenotyping showing decreased class switched B lymphocytes and reduced switched memory B lymphocytes in a case of CD40 deficiency. (A) Control (B) Patient. Lymphocytes were gated on FSc. vs SSc, further B lymphocytes were gated on CD19+ to analyze the B cell subsets. [file Image_6.TIF]

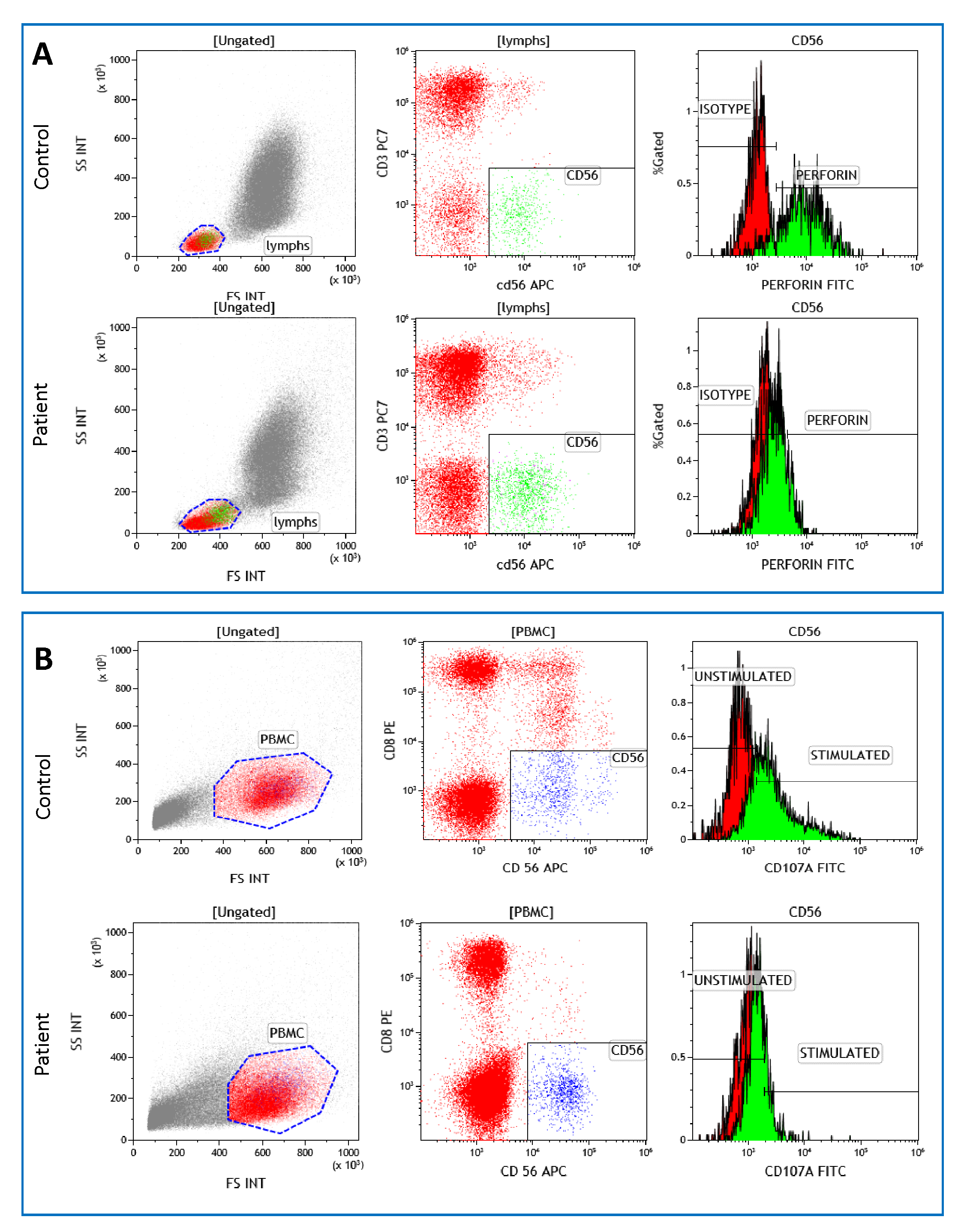

Supplement: Supplementary Figure 7 — Evaluation for hemophagocytic lymphohistiocytosis. (A) Reduced perforin expression on CD56+NK cells in a patient with HLH; lymphocytes were gated on FSc vs. SSc graph. Further, the CD56+ cells are analyzed on gated lymphocytes. (B) PBMCs stimulated with PMA and ionomycin and stained with CD107a show reduced expression in a patient. PBMCs were gated on FSc vs. SSc followed by CD56+ NK cells from the gated PBMCs. [file Image_7.TIF]

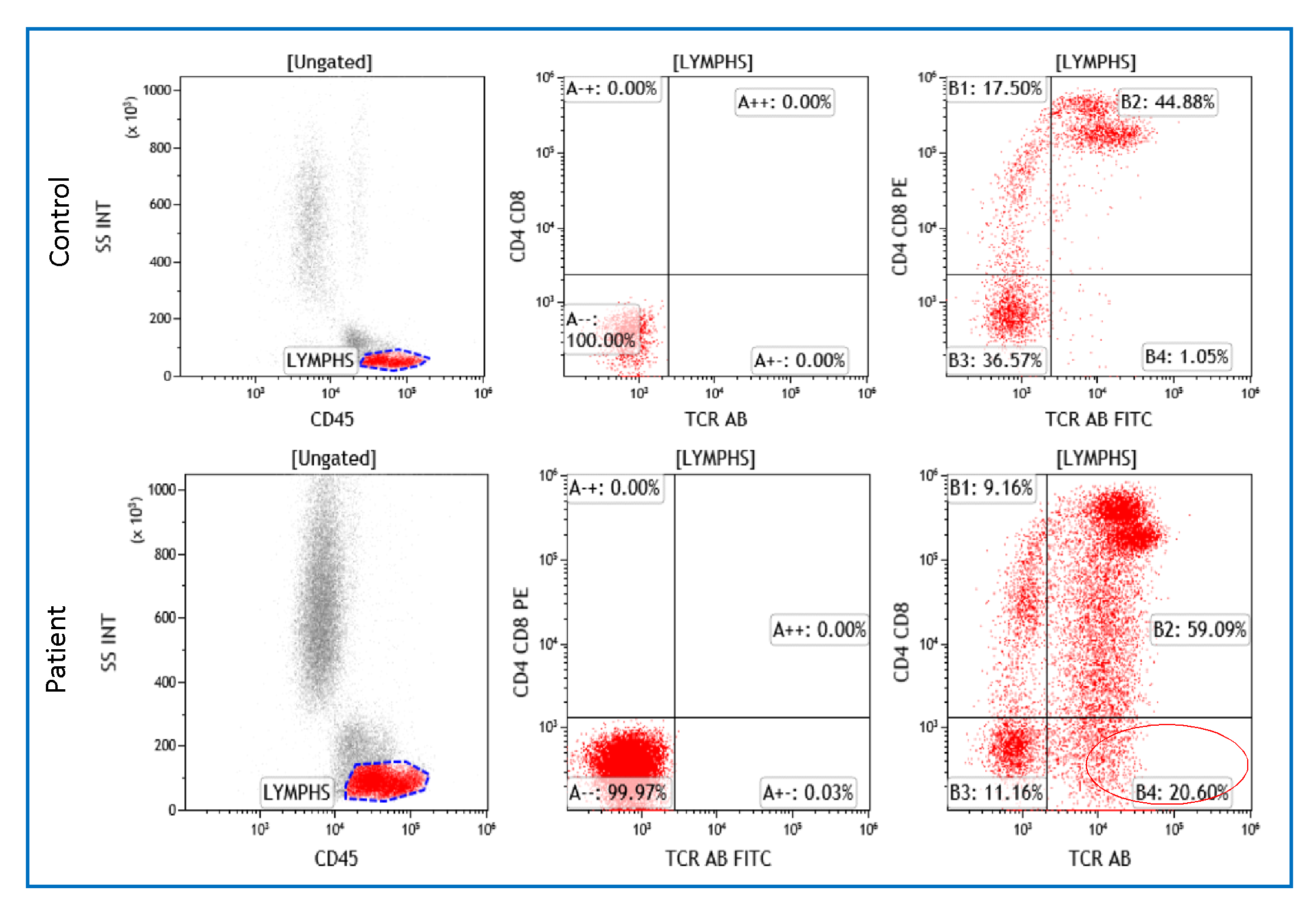

Supplement: Supplementary Figure 8 — Increase in double-negative T cells (TCR αβ+, CD3+, CD4–, CD8–) in a patient with ALPS (FASL mutation). Lymphocytes were gated on CD45 vs. SSc graph and further DNTs were seen on CD4 CD8 vs. TCR-αβ dot plot. [file Image_8.TIF]

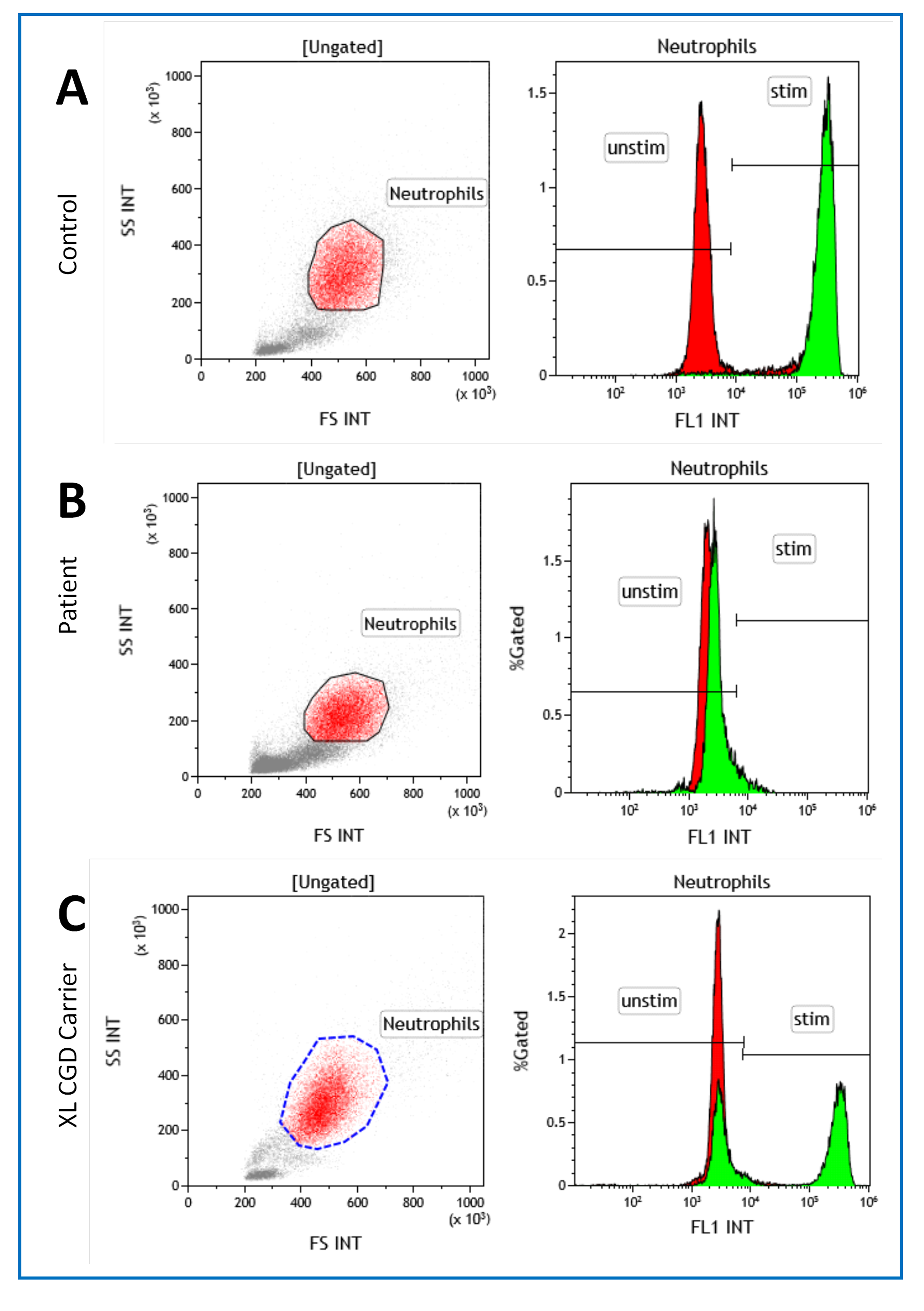

Supplement: Supplementary Figure 9 — Dihydrorhodamine assay for chronic granulomatous disease: (A) Normal shift of fluorescence peak in a control. (B) No shift in a patient with CGD. (C) Bimodal expression in a female carrier of X-linked CGD. Neutrophils were gated on FSc vs. SSc plot and oxidative burst was evaluated on gated neutrophils. [file Image_9.TIF]

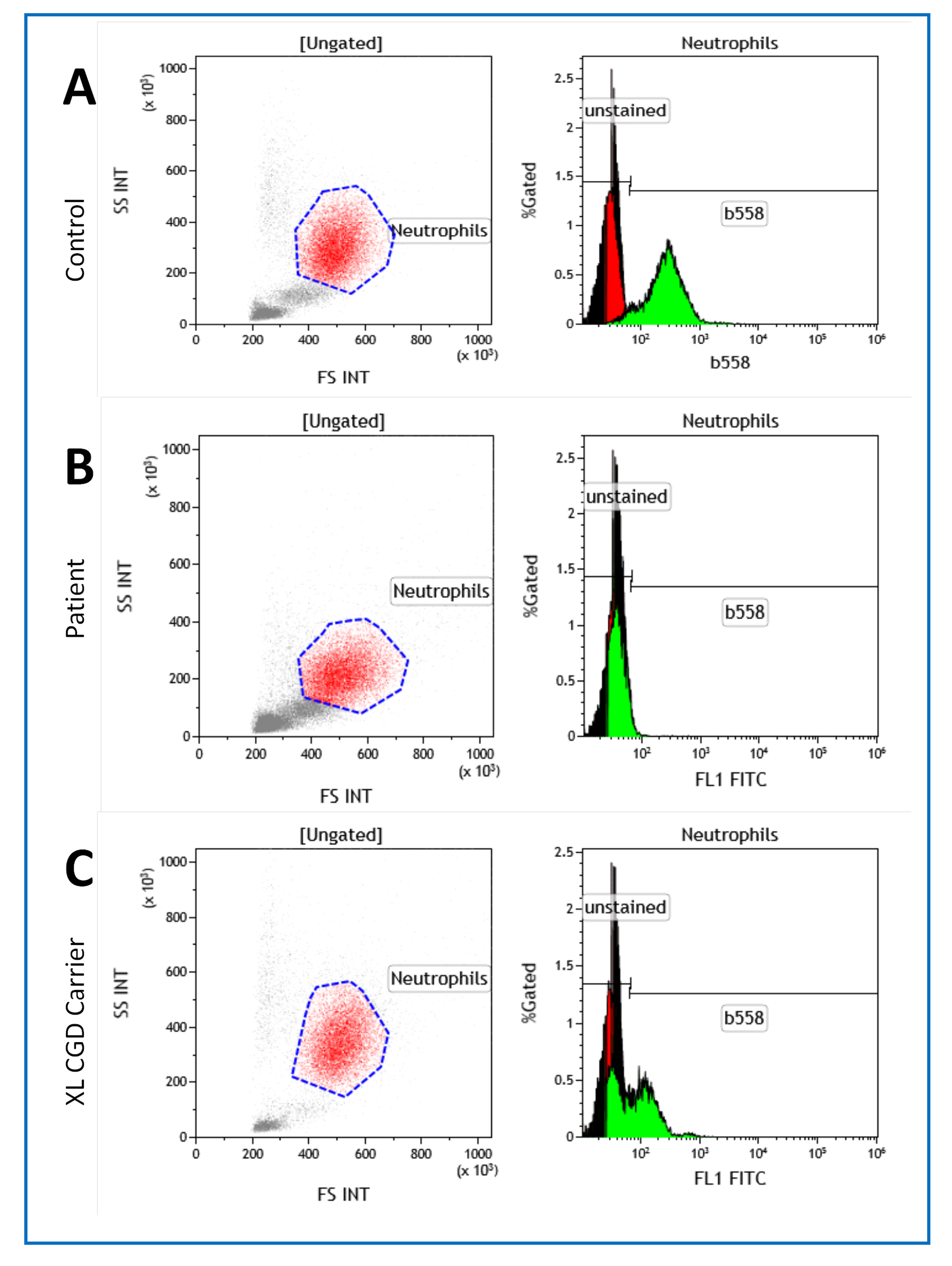

Supplement: Supplementary Figure 10 — Flow cytometry analysis of b558 expression. (A) Normal expression in a control. (B) Patient with X-linked CGD. (C) Dual peaks in a carrier female. Neutrophils were gated on FSc vs. SSc plot and b558 was evaluated on gated neutrophils. [file Image_10.TIF]

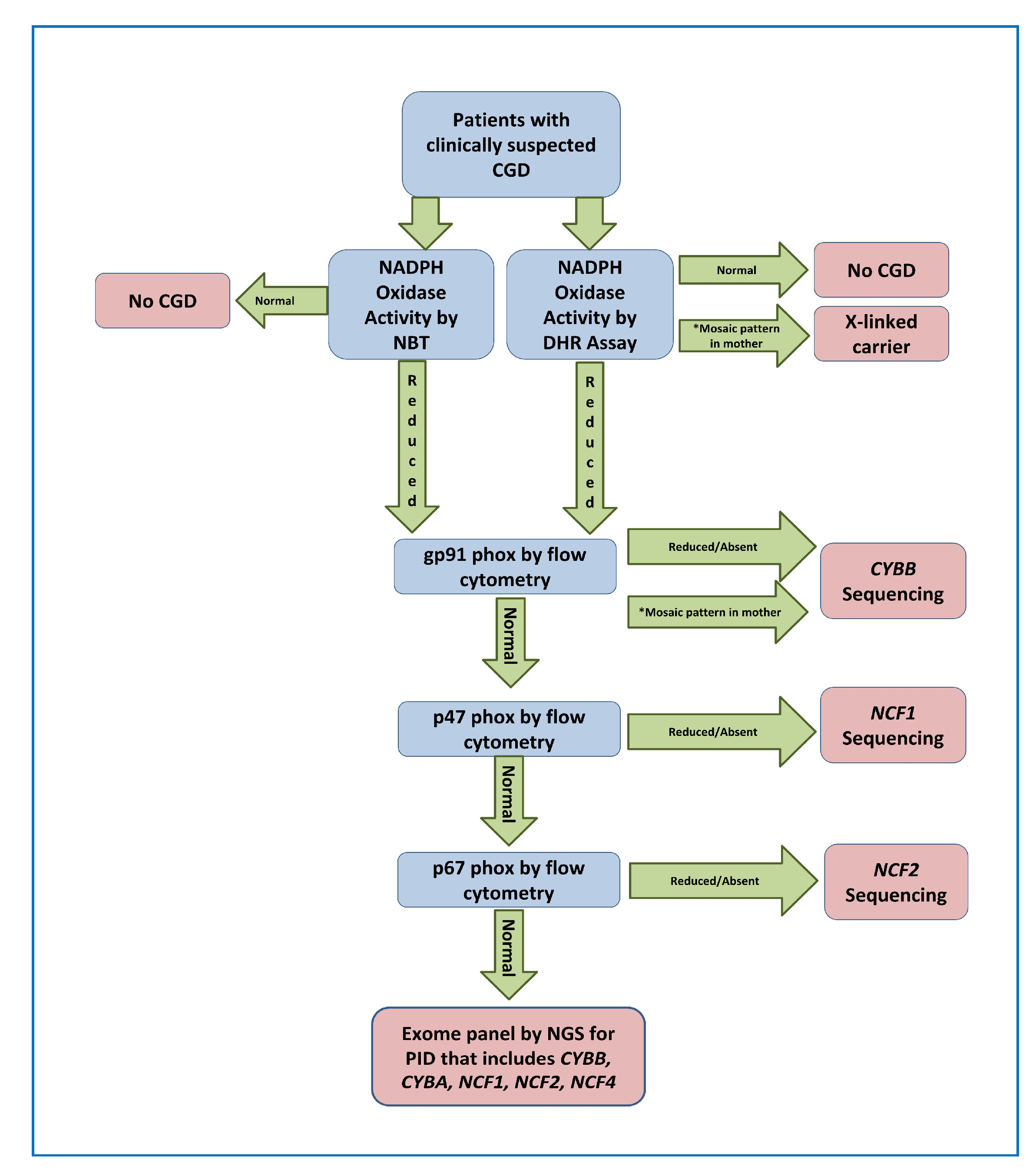

Supplement: Supplementary Figure 11 — Laboratory workflow for patients with chronic granulomatous disease (CGD) diagnosed at our center. *DHR/gp91phox expression in the mother is performed only in cases of males with CGD. [file Image_11.TIF]

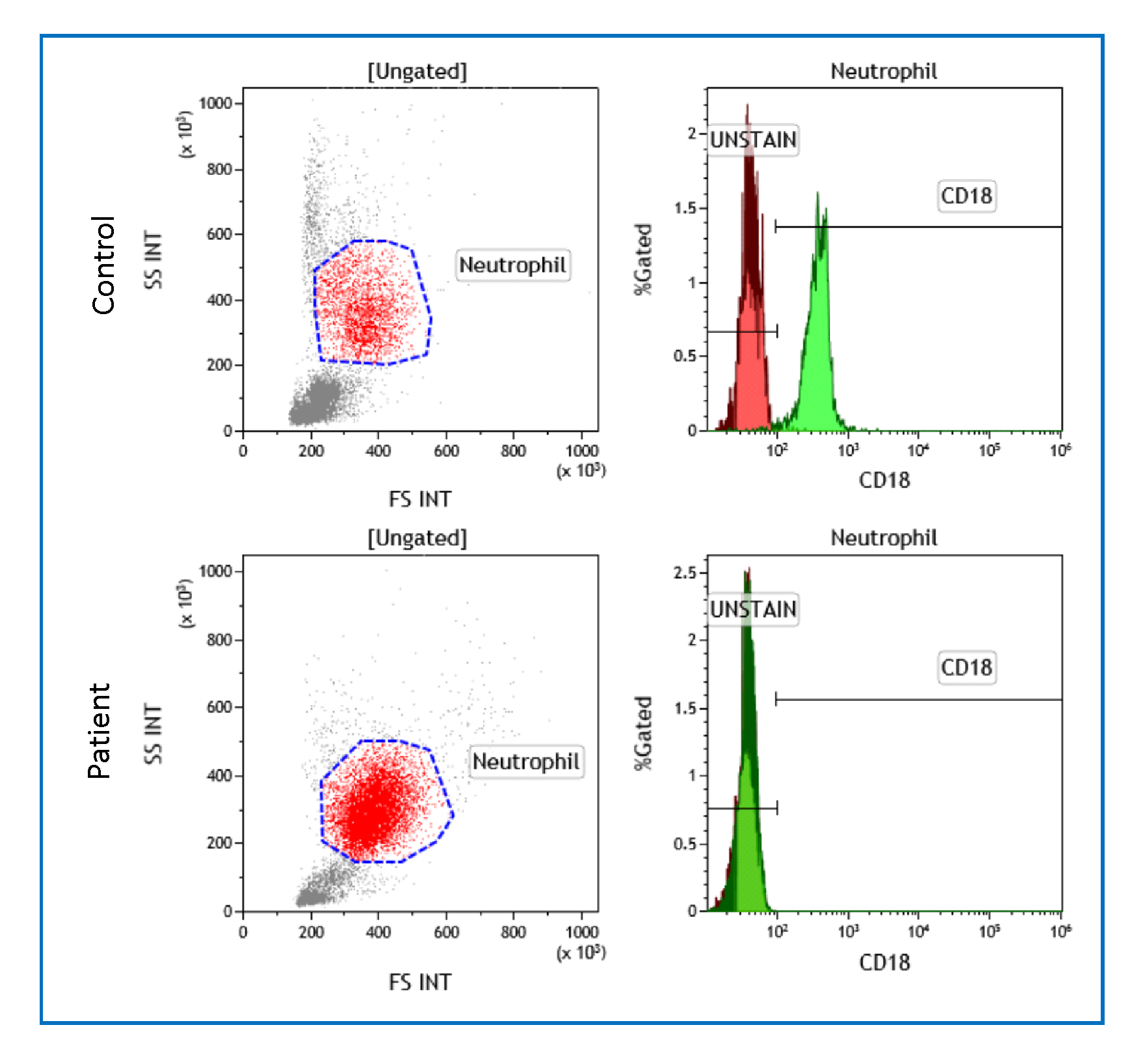

Supplement: Supplementary Figure 12 — Absent expression of CD18 on gated neutrophils in a patient with LAD-1. Neutrophils were gated on FSc vs. SSc plot. [file Image_12.TIF]

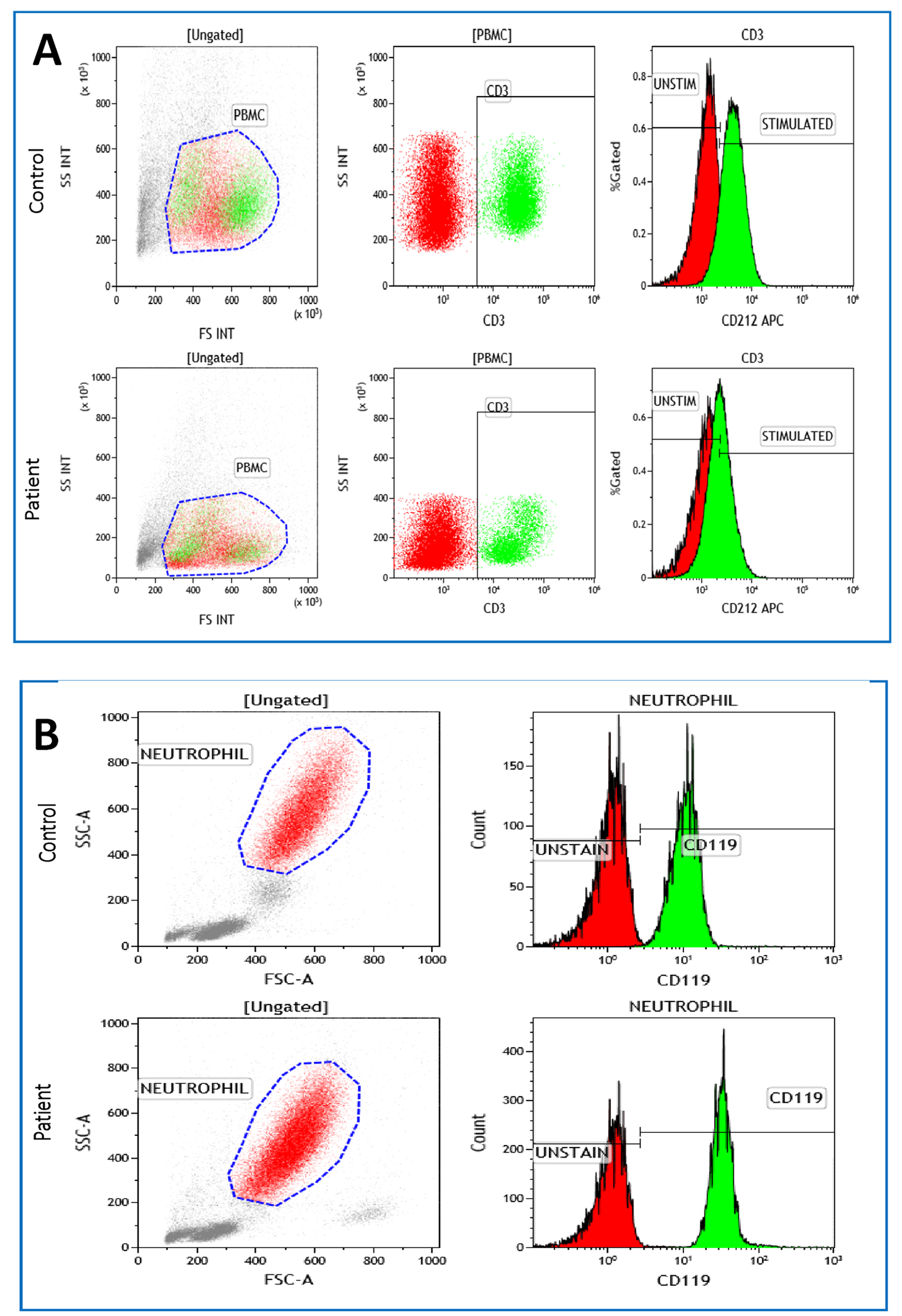

Supplement: Supplementary Figure 13 — (A) Decreased expression of IL12Rβ1 after stimulation of PBMCs with PHA; PBMCs were gated on FSc vs. SSc followed by CD3+ T cells from the gated PBMCs. (B) Increased expression of IFNγR1 expression on gated neutrophils in a patient with partial dominant IFNγR1 defect. Neutrophils, monocytes, and lymphocytes were gated on FSc vs. SSc plot. [file Image_13.TIF]
