# Supplementary material for: Flow Cytometry for Diagnosis of Primary Immune Deficiencies—A Tertiary Center Experience From North India
Source: Front Immunol. 2019 Sep 11;10:2111. doi: 10.3389/fimmu.2019.02111 (PMC6749021; doi:10.3389/fimmu.2019.02111)
Supplement: Supplementary Table 1 — Flow cytometry-based tests for diseases of immune dysregulation. [file Table_1.DOCX]

**Supplementary Table 1. Flow cytometry-based tests for diseases of immune dysregulation**

| Defect | Laboratory Test | Interpretation |
| --- | --- | --- |
| FHLH-2 | Perforin expression | Decreased expression of intracellular perforin on NK cells and CD8+ CTLs |
| FHLH 3, 4, 5 | NK cell Degranulation Assay (CD107a expression) | Decreased/absent expression of CD107a on CD56+ NK cells after stimulation with PMA and ionomycin (41-43) |
| ALPS | Double Negative T cells | Elevated TCRαβ+ CD4-CD8- T cells in blood; (48)  Decreased apoptosis by AnnexinV-PI staining (49) |
| Chediak-Higashi syndrome | NK cell Degranulation Assay (CD107a expression) | Decreased/absent expression of CD107a on CD56+ NK cells after stimulation with PMA and ionomycin |
| Griscelli syndrome Type 2 | NK cell Degranulation Assay (CD107a expression) | Decreased/absent expression of CD107a on CD56+ NK cells after stimulation with PMA and ionomycin |
| IPEX | FoxP3 | Decrease in FoxP3+ T regulatory cells (51) |
| CTLA-4 deficiency | CTLA4 Expression | Decreased CTLA4 expression on T regulatory cells |
| XLP1 | SAP | Decreased expression of SAP in lymphocytes |
| XLP2 | XIAP | Decreased expression of intracellular XIAP in lymphocytes (46) |
| LRBA deficiency | CTLA4 Expression | Decreased CTLA4 expression on T regulatory cells |
|  | LRBA Expression | Decreased expression of intracellular LRBA expression on PBMCs |
| STAT3 gain of function | Double negative T cells  Regulatory T lymphocytes  pSTAT5 and pSTAT1 | Double negative T cells are elevated  Decreased number of regulatory T cells  Impaired STAT5 and STAT1 phosphorylation |
| APECED | Th17 cells | Decreased Th17 cells |

FHLH- Familial Hemophagocytic Lympho-Histiocytosis; ALPS- Autoimmune Lympho-Proliferative Syndrome; IPEX- Immune-dysregulation Polyendocrinopathy Enteropathy X-linked; CTLA4- Cytotoxic T Lymphocyte Associated protein 4; XLP- X-linked Lymphoproliferative disease; LRBA- Lipopolysaccharide Responsive Beige-like Anchor protein; STAT- Signal Transducer and Activator of Transcription; APECED- Autoimmune Polyendocrinopathy Candidiasis Ectodermal Dystrophy; CTL- Cytotoxic T lymphocyte; PMA- Phorbol Myristate Acetate; PI- Propidium iodide; SAP- SLAM Associated Protein; XIAP- X-linked Inhibitor of Apoptosis; PBMC- Peripheral Blood Mononuclear Cell
