# Supplementary material for: Flow Cytometry for Diagnosis of Primary Immune Deficiencies—A Tertiary Center Experience From North India
Source: Front Immunol. 2019 Sep 11;10:2111. doi: 10.3389/fimmu.2019.02111 (PMC6749021; doi:10.3389/fimmu.2019.02111)
Supplement: Supplementary Table 2 — Flow cytometry-based tests for defects in intrinsic and innate immunity. [file Table_2.DOCX]

**Supplementary Table 2. Flow cytometry-based tests for defects in intrinsic and innate immunity**

| Defect | Laboratory Test | Interpretation |
| --- | --- | --- |
| STAT1 GOF | STAT1 dephosphorylation | Decreased Th17 numbers and delayed dephosphorylation of STAT1 after stimulation with IFNγ (4) |
| IL17F deficiency | IL17F | Decrease expression of IL17F on lymphocytes and monocytes after stimulation with PMA and ionomycin |
| IL17RA deficiency | IL17RA | Decrease expression of IL17RA on lymphocytes and monocytes |
| IRAK4 deficiency | Intracellular TNFα | Decreased expression of TNFα in monocytes after stimulation with LPS |
|  | CD62L Shedding Assay | Impaired L selectin shedding on granulocytes (68) |
| MyD88 deficiency | Intracellular TNFα | Decreased expression of TNFα in monocytes after stimulation with LPS (68) |
| MSMD | IFNγR1 | Low/absent expression of CD119 (64)  Decreased pSTAT1 phosphorylation upon stimulation with IFNγ |
|  | IFNγR2 | Low/absent expression of IFNγR2  Decreased pSTAT1 phosphorylation upon stimulation with IFNγ |
|  | CD212 (IL12Rβ1) expression after stimulation | Low/absent expression of CD212 after stimulation with PHA (63)  Decrased STAT4 phosphorylation upon stimulation with IL12 |
|  | STAT1 LOF | Low/absent phosphorylation of STAT1 after stimulation with IFNγ (66) |

CMC- Chronic Mucocutaneous Candidiasis; IRAK4- Interleukin-1 Receptor Associated Kinase 4; MyD88- Myeloid Differentiation primary response 88; MSMD- Mendelian Susceptibility to Mycobacterial Diseases; TNF- Tumor Necrosis Factor; STAT- Signal Transducer and Activator of Transcription; LOF- Loss of function; PMA- Phorbol Myristate Acetate
